# Supplementary material for: Strategy-based cognitive rehabilitation for childhood brain tumour: Protocol for an acceptability and feasibility trial of the Fatigue, Learning, and Memory Enrichment (FLaME) intervention
Source: NIHR Open Res. 2025 Jun 24;5:23. Originally published 2025 Mar 13. [Version 2] doi: 10.3310/nihropenres.13859.2 (PMC12413617; doi:10.3310/nihropenres.13859.2)
Supplement: Peer review history

# Reviewer report: Reviewer response for version 2

**DOI:** 10.3310/nihropenres.15248.r36582

**Published:** 2025-09-12

**Authors:** Ailish Malone

**Affiliations:** 1 RCSI University of medicine and health sciences, Dublin, Ireland

This is a much-needed, rigorously planned and comprehensive study of feasibility and acceptability of a novel rehabilitation intervention for cognitive fatigue in children 7-17 years who have been diagnosed with a brain tumour. The paper makes a clear and convincing case for the need to evaluate acceptability of this intervention, due to the nuances of cognitive impairment and fatigue after a childhood brain tumour and the fact that other interventions have either been burdensome or haven’t reckoned with the unique impact of fatigue. The method of a feasibility trial is appropriate and the aims / objectives map mostly clearly to the detail in the methods. The study has been constructed with best evidence and public involvement, and adheres to rigorous standards for a feasibility trial.

 I commend the authors for their work and I wish them well with this study.

 I see this paper has been reviewed by three colleagues already and the authors have made the requested revisions. I offer the following points as suggestions / clarifications:

 1. For me, the sections in introduction “Theoretical approach to acceptability” and “theoretical approach to outcome measurement” were out of sync in their placement before the study aims and objectives. I think it would read more clearly if these moved to Methods, after stating the objectives.

 2. Objectives 1-5 are mostly clearly addressed with the proposed methods. I wondered about objective 6, “To identify any practical barriers to conducting an RCT intervention at the designated NHS site.” Is this in relation to the intervention taking place at GOSH as distinct from another setting, e.g., community care? How will this be evaluated, e.g., interview?

 3. Consent

- Inclusion criteria point 7 – it is good that capacity to consent will be assumed but where cognitive impairment is severe enough that there is a question about capacity (for older participants who can consent in their own right), what decision supports are in place? This is important for all studies but particularly when the intervention is cognitive rehabilitation, as young people with moderate to severe cognitive impairment could stand to benefit.
- Consent section - The last sentence “For adolescent participants who can provide informed consent or where a parent consenting for their child loses capacity, the participant will not continue in the study and only de-identified data will be retained.” – If the adolescent participant indicates assent, could it be possible to identify another parent or responsible adult who could support their consent? (I realise these scenarios are unlikely.)

4. Recruitment: do you have an indicator of the feasibility of the sample size, e.g., how many children with a diagnosis of a CNS brain tumour attend GOSH annually? Over what timeframe will recruitment be open (feasibility measures – demand – mentions a specific study recruitment window but I don’t see it specified)? Do you have criteria to stop recruitment to the trial? I note the adverse event and serious adverse event protocol and agree with the judgment of low risk of such events, but there may still be red-amber-green criteria worth considering based on slower than anticipated recruitment or low numbers of eligible patients.

 5. Eligibility: I note the inclusion criteria requires a threshold of severity on neuropsychological scales and fatigue scales and that these are done as part of usual care. To clarify, do all children with CNS tumours attending GOSH routinely complete these assessments, or only those referred to neuropsychology based on clinical indication? If the latter, is there a risk that some eligible children could be missed?

 6. PPI – it’s great to see public involvement from multiple approaches and throughout the project.

 7. Intervention – The intervention is described in detail and has been rigorously developed from published evidence and clinical expertise. The terms used to describe the groups differ slightly throughout the paper, e.g., Figure 1 describes “Arm A: intervention (cognitive rehabilitation with fatigue management)” and “Arm B: intervention (cognitive rehabilitation only)”, whereas in the text, subheading “intervention” uses the terms “FLaME programme”, “full intervention” and “cognitive rehabilitation-only intervention”. Is the FLaME programme the novel intervention and only delivered in Arm A / “full” intervention? If so, for clarity I suggest referring to this group as the FLaME intervention group throughout, to be clear that it is the novel intervention. This could be made even clearer by creating a graphic showing the extent to which the groups overlap and differ (this could have value in your participant information sheets too).

 8. Data collection

 TFA – I commend the rigorous development of this study’s TFA questionnaire.

 Table 1 is most helpful in explaining the outcome measurement procedures. All instruments have been rigorously chosen and have value, though I wonder about the time this will take for children and parents during an already busy intervention. This may become evident throughout the study, both in feasibility indicators and the exit interviews, and I encourage the team to consider the burden for a full-scale trial.

 I note objective 4 – “To identify the optimal outcome measures for a larger scale RCT” – do you have pre-defined criteria based on feasibility / acceptability findings for choosing these optimal measures? (This can also be a judgement at the end, upon integration of quant / qual findings).

 9. Data processing and analyses, quantitative data – for such a small sample size, I wonder if there is merit in a purely descriptive approach to statistical analysis, instead of the planned inferential approach with ANOVAs etc. I would be more interested in whether the changes exceeded MCID, for example, than the p value. You can still use the data to power a larger study.

 Congratulations on constructing this important study.

Is the study design appropriate for the research question?

Yes

Is the rationale for, and objectives of, the study clearly described?

Yes

Are sufficient details of the methods provided to allow replication by others?

Yes

Are the datasets clearly presented in a useable and accessible format?

Yes

Reviewer Expertise:

Physiotherapy, rehabilitation, childhood-onset disability, neuro-oncology

I confirm that I have read this submission and believe that I have an appropriate level of expertise to confirm that it is of an acceptable scientific standard.

# Reviewer report: Reviewer response for version 2

**DOI:** 10.3310/nihropenres.15248.r36576

**Published:** 2025-09-05

**Authors:** Florien Boele

**Affiliations:** 1 University of Leeds, Leeds, UK

This is a protocol for a well-designed feasibility trial aiming to enhance cognitive rehabilitation for children with a brain tumour diagnosis.

**Answers to the NIHR Open Research questions**: The rationale and objectives are clearly described and the design is appropriate for the research question. The details on methods and the interventions are described, but not to the extent that others might be able to replicate the study. However, that would be hard to do with two complex interventions being offered – to replicate the study, all intervention materials would need to be provided which is not appropriate for this protocol publication.  

 I do have some other comments the authors might consider to enhance the report, listed below:

**-Abstract:** here, define what cognitive fatigue is? It is explained in the plain language summary but not the scientific abstract.

**-Study objectives**: ‘ *3. To measure preliminary/limited-efficacy patient reported benefit and outcomes of the intervention arms relative to standard care*.’ Above, the theoretical approach to outcome measurement is explained, but up to this point the actual outcomes included in the study are not clear. I would advise to clarify the outcomes you will measure in objective 3.

**-Pre-study PPI**: ‘ *The study was also informed by a recent North Thames survey ^11^ of forty-five families of childhood survivors of PBT where only 2% reported being able to access dedicated cognitive rehabilitation support, and 69% stating they either definitely would have liked their child to receive it (47%) or were unsure (22%).*’ Explain how this has impacted on the study design?

**-Consent**: ‘ *For adolescent participants who can provide informed consent or where a parent consenting for their child loses capacity, the participant will not continue in the study and only de-identified data will be retained.*’ Will this be assessed? How would it be known if a participant or their parent loses capacity?

**-Intervention**: Would add subheadings to clearly indicate the different interventions delivered in each arm (with Arm A receiving both FLaME and cognitive rehab interventions, arm B only cognitive rehab, and arm C standard care). Standard care should also be described in a separate paragraph, as there is a lot of variability in what standard care looks like for this patient group, even across NHS settings.

**-Acceptability measures**: the method described to determine the validity of items makes sense for the questionnaires, but not for qualitative interviews. Can you confirm/explain whether this method was truly applied to the interview topic guides?

**-Preliminary outcome measures**: can you indicate, where applicable, which scores you intend to use for the outcome measures listed? Total scores, specific (sub)scale scores?

**-Data analysis**: quantitative data. Here you state that repeated measures ANOVA will be used. Probably good to clarify what will be done if scores are not normally distributed.

Is the study design appropriate for the research question?

Yes

Is the rationale for, and objectives of, the study clearly described?

Yes

Are sufficient details of the methods provided to allow replication by others?

Partly

Are the datasets clearly presented in a useable and accessible format?

Not applicable

Reviewer Expertise:

adult neuro-oncology, family caregiving, quality of life and cognitive outcomes, clinical trials

I confirm that I have read this submission and believe that I have an appropriate level of expertise to confirm that it is of an acceptable scientific standard, however I have significant reservations, as outlined above.

# Reviewer report: Reviewer response for version 1

**DOI:** 10.3310/nihropenres.15060.r34982

**Published:** 2025-04-08

**Authors:** Ingrid Tonning Olsson, Jonas Lödén

**Affiliations:** 1 Lund University, Lund, Sweden; 2 Paediatrics, Enhet kurator psykolog barn, Skanes universitetssjukhus Lund, Lund, Skåne County, Sweden

This is a study protocol for an acceptability and feasibility trial of a strategy-based cognitive rehabilitation intervention for survivors of pediatric brain tumors, an area of research where well-designed studies is severely needed. The proposed study has a well worked-through design with three treatment arms: cognitive rehabilitation and fatigue intervention, cognitive rehabilitation only, and standard care. Strengths of the study are the combination of both theoretical and clinical knowledge, and the inclusion of comprehensive patient and public involvement when developing the intervention. Another strength is the inclusion of several good measures of feasibility.

**Is the rationale for, and objectives of, the study clearly described?**

 The authors argue that many previously researched interventions for these patients have had a drill-based, reductionistic focus with low completion rates. They aim to address this by designing a more comprehensive intervention that targets both cognitive late effects and fatigue. Finding factors associated with successfully conducted strategic rehabilitation interventions is of utmost importance in strengthening the quality of life for survivors.

 The objectives of the study are clearly described, and the background is comprehensive and clear. The overall objective of the proposed study is to establish feasibility and acceptability for a strategy-based cognitive rehabilitation intervention, and this is further broken down into six different sub-aims in a very clear and consistent way.

**Is the study design appropriate for the research question?**

 Strengths of the study design are a solid and comprehensive patient and public involvement during the development of the intervention, a very well-designed assessment of feasibility and acceptability, and the use of individualized rehabilitation goals. These methods are appropriate for answering research questions. The proposed intervention combines previously tested intervention methods used for fatigue and acquired brain injury for children, although untested in this population.

 A convenience sample is used, meaning that the study might suffer from bias, for example related to ethnicity or tumor type. Having received a neuropsychological evaluation at Great Ormond Street Hospital (GOSH) is an inclusion criterion, but it is not described what the criteria are for such an evaluation. Of course, a stratified sample would be preferable for this aim, but it is likely not possible.

 A comment on why the cut off-variables are different for fatigue and cognitive sub domains would be helpful – why the authors apply different cut-offs for impairment: z<-.67 for fatigue and z<-1 for cognition.

 One of the aspects missing in the analysis is the comparison of participants receiving the intervention remote compared to face-to-face. The authors suggest that this can be done without compromising the fidelity or efficacy of the intervention. Even though this is likely, given previous research regarding remote interventions of cognitive rehabilitation, an analysis of this would be helpful in future tailoring of the intervention and of importance when conducting the following RCT.

 Another strength of the study is the theoretical framework of the pediatric neurocognitive intervention model, ^1^ stating that every intervention needs to start with addressing psychosocial and systemic needs first, e.g., a family with a very heavy workload or a family with inadequately treated mental illnesses, might not be able to benefit from rehabilitation. I cannot see that this is addressed in the intervention program, e.g., by administering the Psychosocial Assessment Tool ^2^ before starting the intervention. Since psychosocial obstacles might be difficult to eliminate or alleviate, such screening might lead to the exclusion of families whose psychosocial status might compromise their participation, which would have benefited the study.

**Are sufficient details of the methods provided to allow replication by others?**

 In an otherwise well-described method section, the listed seven inclusion criteria would benefit from some clarification (and these are very minor remarks, probably typos):

 Criterion 2: The text states that survivors of brain tumors are eligible, but the text says “…and/or oncology treatment to brain”, which might imply inclusion of survivors of leukemia and non-Hodgkin.

 Criterion 3: states that survivors should have received a neuropsychological assessment or be under active surveillance with the multidisciplinary team. The latter is neither defined nor given a cut-off. The neuropsychological assessment is not described, e.g., how many subtest should be included. Given that the probability of having at least one score <=-1z-score is very high if you are taking 10 subtests (about 82%), this is of importance. Also, wording is a bit unclear: “one or more scores outside the normal limits (i.e. 1 SD above or below the mean in the direction indicating difficulty) in at least one neuropsychological domain…”. I guess the authors want to say that any score <=-1SD would be an inclusion criterion. Or do they want to include survivors with very uneven profiles, e.g., those with an average overall performance and one or two very high scores?

 Criterion 6: Impairment=z>-0.67. PedsQL MFS has a scale with higher scores indication less fatigue. I guess it should be z<-0.67 here, or (if the scale is reversed): z<0.67

 Given that the study compares interventions to standard care, a description of what standard care consists of would provide more insight into the differences in being included in one of the other study arms. This also affects the replicability of the study. Given that the study is conducted at a single center, the standard treatment might be specific to that site. A description of the standard treatment would therefore also increase the replicability of the study.

**Are the datasets clearly presented in a useable and accessible format?**

 This is a strength of the proposed study with well-designed graphs and tables depicting the workflow. The different measurement methods are clearly presented. It is easy to follow which data will be collected and in which way it will be analyzed.

**Conclusions**

 This is a well-needed and well-designed study that will fill a research gap, and we are looking forward to the results. The proposed methods are suitable for the aims, the intervention is well described and founded in both clinical and theoretical knowledge, as well as guided by patient and public involvement. Some minor aspects of the method could be clarified. We recommend the authors to:

 1) Clarify of how the interventions differentiate from standard care.

 2) Clarify inclusion criteria and provide a rationale for the proposed cut-off limits.

 3) Include some form of standard screening of psychosocial and systemic factors before the intervention following the pediatric neurocognitive intervention model.

 3) Inclusion of an analysis of differences when the intervention is remote vs on-site.

Is the study design appropriate for the research question?

Yes

Is the rationale for, and objectives of, the study clearly described?

Yes

Are sufficient details of the methods provided to allow replication by others?

Partly

Are the datasets clearly presented in a useable and accessible format?

Yes

Reviewer Expertise:

Neuropsychology, neurocognitive late complication following childhood cancer

We confirm that we have read this submission and believe that we have an appropriate level of expertise to confirm that it is of an acceptable scientific standard.

## References

[1] : A model for pediatric neurocognitive interventions: considering the role of development and maturation in rehabilitation planning. *Clin Neuropsychol* .2014;28(2) : 10.1080/13854046.2013.873083 181-98 10.1080/13854046.2013.873083 24498935

[2] : Pediatric psychosocial preventative health model: Achieving equitable psychosocial care for children and families. *Fam Syst Health* .2024;42(1) : 10.1037/fsh0000856 76-89 10.1037/fsh0000856 38010765

# Response

**Published:** 2025-06-21

**Authors:** Charlotte P. Malcolm

**Affiliations:** Neuropsychology Service, Great Ormond Street Hospital for Children NHS Foundation Trust, London, London, UK

AUTHOR RESPONSE: Thank you very much for reviewing the manuscript and for the positive comments which we were very pleased to receive. We were particularly pleased to hear the positive feedback on the design of the study. We fully agree with the important need to develop strategic rehabilitation interventions for childhood brain tumour survivors for strengthening quality of life, and we hope this research trial will add useful evidence towards this. We look forward to sharing the results from the study. This is a study protocol for a feasibility trial which will inform the design of a future Randomised Controlled Trial (RCT). The feasibility trial allows us to trial certain aspects of the design to inform the RCT.

  We provide clarifications as requested below:

**REVIEWER COMMENT: This is a well-needed and well-designed study that will fill a research gap, and we are looking forward to the results. The proposed methods are suitable for the aims, the intervention is well described and founded in both clinical and theoretical knowledge, as well as guided by patient and public involvement. Some minor aspects of the method could be clarified. We recommend the authors to:**

**REVIEWER COMMENT: 1) Clarify of how the interventions differentiate from standard care.**

 AUTHOR RESPONSE: We have now clarified standard care in the manuscript on pages 10-11 (‘Study design and setting’) as:

 “ *Standard care at the study site consists of 1) a single feedback session on the neuropsychological assessment findings and recommendations, and 2) continuation of routine oncology medical care, as appropriate for the patient, without any neuropsychological intervention”.*

 The primary difference between standard care and treatment arms in the study is that patients will receive a neuropsychological intervention in the trial, which is not available within the current service for children with brain tumour.

**REVIEWER COMMENT: 2) Clarify inclusion criteria and provide a rationale for the proposed cut-off limits.**

 AUTHOR RESPONSE: Clarification for each criterion requested is provided below. Please note that eligibility criteria have been peer-reviewed and approved by a research ethics committee in advance of opening the trial. The eligibility criteria will be evaluated as part of the feasibility trial to inform a definitive RCT.

  

- **REVIEWER COMMENT: Criterion 2: The text states that survivors of brain tumors are eligible, but the text says “…and/or oncology treatment to brain”, which might imply inclusion of survivors of leukemia and non-Hodgkin.**

AUTHOR RESPONSE: This criterion was to include patients with brain tumour and those who have head and neck tumours (e.g., rhabdomyosarcoma) with radiation fields to the brain who access the Neuropsychology service. These children present with a similar level of cognitive deficit and late effects to those with a primary brain tumour on account of radiation doses to the brain. These patients are very rare (0-2 cases referred per year). On ethical review we could not exclude these patients given their cognitive needs which have the same aetiology as children with primary brain tumours. We will describe the clinical demographics of the final sample as part of the feasibility trial. Survivors of leukaemia and non-Hodgkin do not access our service and therefore will not be recruited into the trial. We have provided clarification in the manuscript about our Neuropsychology service eligibility criteria as below (pg. 18, ‘Sampling and sample size’):

 “ *All children with a diagnosis of brain tumour under GOSH Neuro-Oncology medical care can be referred to the Neuropsychological service on a combination of prospective and bespoke protocols.  Children treated curatively for high-grade solid tumours (including head and neck tumours with brain radiation), or low-grade brain tumours and radiotherapy,  are assessed at baseline, 2 years, and 5 years post treatment. Children with low-grade tumours without radiation are referred based on known risk factors for cognitive impairment (e.g., shunted hydrocephalus, midline tumours) and/or presenting cognitive concerns.”*

  

- **REVIEWER COMMENT: Criterion 3: states that survivors should have received a neuropsychological assessment or be under active surveillance with the multidisciplinary team. The latter is neither defined nor given a cut-off. The neuropsychological assessment is not described, e.g., how many subtest should be included. Given that the probability of having at least one score <=-1z-score is very high if you are taking 10 subtests (about 82%), this is of importance. Also, wording is a bit unclear: “one or more scores outside the normal limits (i.e. 1 SD above or below the mean in the direction indicating difficulty) in at least one neuropsychological domain…”. I guess the authors want to say that any score <=-1SD would be an inclusion criterion. Or do they want to include survivors with very uneven profiles, e.g., those with an average overall performance and one or two very high scores?**

AUTHOR RESPONSE: We have now added to pg. 18 (‘Sampling and sample size’) that “ *All children treated for brain tumour remain under surveillance with the multidisciplinary team until they transition to adult services* *”.* We have now also included details of the cognitive tests included in a typical neuro-oncology neuropsychology assessment battery as supplementary extended data to the manuscript ( https://doi.org/10.6084/m9.figshare.29376176, referenced on page 18 ‘ *Neuropsychological assessment data*’). As described in the eligibility criteria, the focus of eligibility is on cognitive measures (i.e. not visual-motor). We intentionally kept the inclusion open to explore feasibility and it is consistent with previous cognitive rehabilitation studies in this population (references 11 and 12). Given the paucity of research, it is unclear which children would benefit from intervention and it is important to acknowledge that we are working with a condition where emerging cognitive late effects are very common and could potentially be mitigated with earlier intervention. We trialled our inclusion criteria in advance of our ethics review submission and found that it was very rare to have only one or two scores an SD below the mean. In only 1 of 57 cases (1.7%) did a child have only one score of 1 SD below the mean and they reported associated functional difficulties with this that would benefit from intervention. In most cases, if a child had one score in this range, this was accompanied by several other scores in this range across multiple cognitive domains. The cognitive profile of the feasibility sample recruited will be described with the feasibility study findings.

  

- **REVIEWER COMMENT: Criterion 6: Impairment=z>-0.67. PedsQL MFS has a scale with higher scores indication less fatigue. I guess it should be z<-0.67 here, or (if the scale is reversed): z<0.67.**

AUTHOR RESPONSE: We confirm that where z>-0.67 PedsQL MDFS is stated, this is indicating the direction below the mean (0) where a minus score indicates greater difficulty.

**REVIEWER COMMENT: A comment on why the cut off-variables are different for fatigue and cognitive sub domains would be helpful – why the authors apply different cut-offs for impairment: z<-.67 for fatigue and z<-1 for cognition.**

 AUTHOR RESPONSE: We used a cut-off of 1 SD above or below the mean for cognition as this is conventional and has precedence for neuropsychological measures. As there is no similar convention or standardisation for fatigue symptoms using the MDFS in this population, we selected a more clinical and conservative cut-off score that indicates the ‘below average’ range, indicating children who might benefit from the intervention.

**REVIEWER COMMENT: 3) Include some form of standard screening of psychosocial and systemic factors before the intervention following the pediatric neurocognitive intervention model.**

 AUTHOR RESPONSE: There are two elements to this point which relate to the PNI model and psychosocial factors. Firstly, whilst we drew on aspects of the PNI model, the intervention was not designed to follow it exactly. We developed the FLaME intervention to include a common co-morbidity in paediatric brain injury (i.e. fatigue) as a foundational factor in the intervention. Therefore, the FLaME intervention is a bespoke adaptation of the model for the study. Secondly, we agree that psychosocial factors are important to consider but they ae separate to the content of the FLaME trial. Within the healthcare system of this study, psychosocial factors are independently screened by a multidisciplinary team and referred for intervention to a Clinical Psychology and Social Work team rather than the Neuropsychology service, which leads this trial. As part of our trial screening visit, we document other psychological interventions the child and family may have received in the 6 months prior to the trial and we will describe this as part of the feasibility assessment to inform consideration for the definitive RCT. We also document any psychosocial factors given as a reason for declining or withdrawing from the study as part of the feasibility analysis to inform the RCT.

**REVIEWER COMMENT: 4) Inclusion of an analysis of differences when the intervention is remote vs on-site.**

 AUTHOR RESPONSE: We agree this will be beneficial and is included in the analyses plan for the feasibility and acceptability measures, if possible. Feasibility trials are not intended to generate sufficient quantitative data to statistically compare outcomes for efficacy, which is instead the aim of the definitive RCT. We will include descriptive analysis of the outcome measures between remote and in-person delivery, if there are sufficient data and variability in modality to do this.

 With many thanks again for the helpful comments.

# Reviewer report: Reviewer response for version 1

**DOI:** 10.3310/nihropenres.15060.r34981

**Published:** 2025-04-08

**Authors:** Jurgen Lemiere

**Affiliations:** 1 Pediatric Oncology,, KU Leuven, Belgium

Given the significant impact of neurocognitive problems on pediatric brain tumor patients and the current lack of effective interventions, this study is of great importance.

 The authors state that up to 100% of children treated for PBT experience some degree of cognitive morbidity. However, the reference used pertains only to PBT located in the Fossa Posterior, which is not fully representative of the broader claim made.

 Additionally, the authors mention that neurocognitive problems increase over time since treatment, with an average loss of 18 FSIQ points by early adulthood. One of the references supporting this claim is over 20 years old, and the 18 FSIQ points drop is not applicable to all types of PBT. Therefore, a more nuanced and up-to-date paragraph on the neuropsychological impact of PBT is warranted.

 The paragraph on neurorehabilitation is missing some potential intervention methods, such as medication and lifestyle changes. I am aware of at least one systematic review on this topic that could be included to provide a more comprehensive overview.

 Regarding cognitive fatigue, the paragraph could benefit from a broader framework on fatigue. Since fatigue is multimodal, the authors should maintain consistency across the manuscript by specifically referring to cognitive fatigue rather than general fatigue.

 A positive aspect of the study is the active involvement of patients and the creation of a Patient Advisory Group (PAG).

 The age range for participants is quite large, spanning from 7 to 18 years. It is important to clarify whether the program is similar for all age ranges, as younger children may require more parental involvement. Additionally, will the randomization process account for the different age ranges in the treatment arms?

 In the inclusion criteria, it is mentioned that an impairment score on one or more subscales of PEDSQL is required. This implies that the study is not solely focused on cognitive fatigue but on general fatigue. While it is challenging to disentangle different aspects of fatigue, the authors should be precise about the domain of fatigue being investigated and ensure coherence between the introduction (cognitive fatigue) and the study's focus (all aspects of fatigue).

 Could the authors provide more details on the neuropsychological assessment and how the scores will be handled? For instance, if multiple scores for a neuropsychological domain are available and these scores are discrepant, how will this be managed? For example, the CPT includes many scores; will all scores be considered?

 Regarding the intervention, the focus is on strategies for cognitive fatigue. Please refer to my previous remarks on this topic.

Is the study design appropriate for the research question?

Yes

Is the rationale for, and objectives of, the study clearly described?

Yes

Are sufficient details of the methods provided to allow replication by others?

Yes

Are the datasets clearly presented in a useable and accessible format?

Yes

Reviewer Expertise:

Neuropsychology in pediatric cancer, behavioural therapy

I confirm that I have read this submission and believe that I have an appropriate level of expertise to confirm that it is of an acceptable scientific standard, however I have significant reservations, as outlined above.

# Response

**Published:** 2025-06-21

**Authors:** Charlotte P. Malcolm

**Affiliations:** Neuropsychology Service, Great Ormond Street Hospital for Children NHS Foundation Trust, London, London, UK

**REVIEWER COMMENT: Given the significant impact of neurocognitive problems on pediatric brain tumor patients and the current lack of effective interventions, this study is of great importance.**

 AUTHOR RESPONSE: Thank you for reviewing the manuscript and for the positive comments on the study which we were very pleased to receive. We were particularly pleased that the importance of rehabilitation studies in this population is recognised.

**REVIEWER COMMENT: The authors state that up to 100% of children treated for PBT experience some degree of cognitive morbidity. However, the reference used pertains only to PBT located in the Fossa Posterior, which is not fully representative of the broader claim made. Additionally, the authors mention that neurocognitive problems increase over time since treatment, with an average loss of 18 FSIQ points by early adulthood. One of the references supporting this claim is over 20 years old, and the 18 FSIQ points drop is not applicable to all types of PBT. Therefore, a more nuanced and up-to-date paragraph on the neuropsychological impact of PBT is warranted.**

 AUTHOR RESPONSE: Reference 3 (below) has been added to support the point about the high prevalence of neurocognitive morbidity for children with a range of brain tumours and associated treatments. The authors review a large body of evidence in support of neurocognitive impairment being the most pervasive late effect.

 Pancaldi A, Pugliese M, Migliozzi C, et al.: *Neuropsychological outcomes of children treated for brain tumors.* Children (Basel). 2023; 10(3): 472.

 We have amended the introductory paragraph to include further nuance on risk factors for neurocognitive impairment supported by citations from 2025 as below:

*Treatment advances for paediatric brain tumour (PBT) in recent decades have substantially improved mortality rates but come at significant cost in cognitive morbidity alongside the long-term effects of the tumour itself. Up to 100% of children treated for PBT experience some degree of cognitive difficulty despite most having achieved typical cognitive development prior to diagnosis ^1^ ^3^. Risk factors for neurocognitive impairment are varied and continue to be investigated. The most consistent risk factors for neurocognitive impairment have included provision and dose of cranial radiation, younger age at treatment, shunted hydrocephalus, and cerebellar mutism syndrome ^3, Horne et al., 2025)^.  Most children experience a constellation of long-term cognitive impairments that impact quality of life, mental health, access to education, academic and vocational attainment, and functional independence in adulthood. The most common neurocognitive impairments are in processing speed, attention, working and long-term memory, and visual-motor function which increase with time since treatment and result in the insidious secondary slowing of intellectual and academic progress over time (neurocognitive ‘late effects’), with reported average loss of 18 Full-Scale IQ points by early adulthood following cranial irradiation ^2^^, ^^3^ . Despite developments in oncology treatment protocols, survivors of common malignant tumours (e.g., medulloblastoma) remain at risk of significant neurocognitive and functional impairment into adulthood ^Papini et al., 2025^.*

 Additional citations:

 Horne, B. M., Attanayake, A. A., Aquilina, K., Murphy, T., & Malcolm, C. P. (2025). The Neurocognitive Profile of Post-operative Paediatric Cerebellar Mutism Syndrome: A Systematic Review. *medRxiv*, 2025-02. doi.org/10.1101/2025.02.21.25322700

 Papini C, Mirzaei S, Xing M, Tonning Olsson I, Salloum R, de Blank PMK, Lange KR, King TZ, Srivastava D, Leisenring WM, Howell RM, Oeffinger KC, Robison LL, Armstrong GT, Krull KR, Brinkman TM. Neurocognitive outcomes and functional independence in adult survivors of childhood medulloblastoma diagnosed over 3 decades. *Neuro Oncol*. 2025 Jan 12;27(1):254-266. doi: 10.1093/neuonc/noae119. PMID: 38963825; PMCID: PMC11726255.

**REVIEWER COMMENT: The paragraph on neurorehabilitation is missing some potential intervention methods, such as medication and lifestyle changes. I am aware of at least one systematic review on this topic that could be included to provide a more comprehensive overview.**

 AUTHOR RESPONSE: As the focus of the study is on cognitive rehabilitation interventions it is beyond the scope of the introduction to discuss in detail other interventions such as pharmacological or exercise interventions. We have provided a citation to the systematic review in case readers wish to read about these interventions in paragraph 2 of the *Introduction*:

 “ *Interventions to mitigate neurocognitive impairment have included lifestyle changes, pharmacological intervention, and cognitive rehabilitation ^Bullens et al., 2024^* *”.*

 Additional citation:

 Bullens, K., Sleurs, C., Blommaert, J., Lemiere, J., & Jacobs, S. (2024). A systematic review of interventions for neurocognitive dysfunctions in patients and survivors of a pediatric brain tumor. *Pediatric Blood & Cancer*, *71*(12), e31327. DOI: 10.1002/pbc.31327

**REVIEWER COMMENT: A positive aspect of the study is the active involvement of patients and the creation of a Patient Advisory Group (PAG).**

 AUTHOR RESPONSE: Thank you.

**REVIEWER COMMENT: The age range for participants is quite large, spanning from 7 to 18 years. It is important to clarify whether the program is similar for all age ranges, as younger children may require more parental involvement. Additionally, will the randomization process account for the different age ranges in the treatment arms?**

 AUTHOR RESPONSE: As described in the manuscript, the intervention is delivered according to a developmental hierarchical model. Given the level of cognitive difficulty typically found in this population we anticipate that parental/adult support will be beneficial for implementing and transferring interventional strategies for all age groups and this is planned for in the intervention. We describe on pg. 12 under (‘ *Intervention’*), that sessions are delivered with the child and their parent/caregiver, with strategies also shared with their teacher. We have further added: “ *The programme content and concepts (e.g., PQRST, chunking, pacing) per session are consistent across age groups, but the delivery (e.g., wording, supporting images, and consolidation activities) is adapted by age. Degree of involvement of an adult in intervention activities can be adapted according to the child’s level of development and independence*”.

 As this is a small n feasibility trial, we do not include any stratified randomisation. The need for this is instead evaluated as a feasibility outcome to inform the definitive RCT which will both focus on and be powered to assess efficacy and effectiveness.

**REVIEWER COMMENT: Regarding cognitive fatigue, the paragraph could benefit from a broader framework on fatigue. Since fatigue is multimodal, the authors should maintain consistency across the manuscript by specifically referring to cognitive fatigue rather than general fatigue. In the inclusion criteria, it is mentioned that an impairment score on one or more subscales of PEDSQL is required. This implies that the study is not solely focused on cognitive fatigue but on general fatigue. While it is challenging to disentangle different aspects of fatigue, the authors should be precise about the domain of fatigue being investigated and ensure coherence between the introduction (cognitive fatigue) and the study's focus (all aspects of fatigue).**

 AUTHOR RESPONSE: This is a complex area with limited sensitivity in measurement. There are few measures of fatigue or cognitive fatigue for the paediatric population, and the sensitivity and specificity of the measures in identifying cognitive fatigue in the paediatric brain tumour population have not been studied. There is an absence of literature on the differences between cognitive and general fatigue, especially for paediatric brain injury and brain tumour population, that limits detailed discussion. We have made this point more explicit in the manuscript on pg. 7 (‘ *Introduction’*) where we also discuss the absence of literature generally on cognitive fatigue in paediatric brain tumour: “ *with very few studies conceptually distinguishing cognitive fatigue from fatigue in general*”. We selected the MDFS as this is the only measure that at least facilitates compartmentalising fatigue, but an important element of the study is to evaluate change in these domains pre- and post- an intervention that focuses on *cognition and cognitive fatigue*. In our clinical experience of this population, sequelae of cognitive fatigue are often reflected across the domains of the MDFS (e.g., problems starting and finishing activities, feeling tired or napping after cognitive activities) but with higher scores within the cognitive domain. We will evaluate this profile in the final sample to inform if and how this measure should be used in an RCT and to guide recommendations for future research for conceptual study of cognitive versus general fatigue in this population. We have added this point to the manuscript in the justification for selecting the MDFS (pages 14-15 ‘ *Primary outcome measures’*) to provide further context on both terminology/conceptualisation and measurement.

**REVIEWER COMMENT: Could the authors provide more details on the neuropsychological assessment and how the scores will be handled? For instance, if multiple scores for a neuropsychological domain are available and these scores are discrepant, how will this be managed? For example, the CPT includes many scores; will all scores be considered?**

 AUTHOR RESPONSE: We have now included details of the cognitive tests included in a typical neuro-oncology neuropsychology assessment battery as supplementary extended data to the manuscript ( https://doi.org/10.6084/m9.figshare.29376176, referenced on page 18 ‘ *Neuropsychological assessment data*’). We will recruit participants according to the stated eligibility criteria of one or more scores 1 SD above or below the mean in the direction indicating difficulty in cognitive domains rather than by discrepancy. We intentionally kept the inclusion open to explore feasibility and it is consistent with the inclusion criteria in previous cognitive rehabilitation studies in this population (citations 11 and 12). Given the paucity of research, it is unclear which children would benefit from intervention and it is important to acknowledge that we are working with a condition where emerging late effects are very common and could potentially be mitigated with earlier intervention. We trialled our inclusion criteria in advance of our ethics review and found that it was very rare to have only one or two scores an SD below the mean. In only 1 of 57 cases (1.7%) did a child have only one score of 1 SD below the mean and they reported associated functional difficulties with this that would benefit from intervention. In most cases, if a child had one subtest or index score in this range, this was accompanied by several other subtest and index scores within and across cognitive domains. The cognitive profile of the feasibility sample recruited will be described with the feasibility study findings.

 Citations:

 11. Butler RW, Copeland DR, Fairclough DL, et al.: A multicenter, randomized clinical trial of a cognitive remediation program for childhood survivors of a pediatric malignancy. J Consult Clin Psychol. 2008;76(3):367–78. 18540731 10.1037/0022-006X.76.3.367 2827251

 12. Patel SK, Katz ER, Richardson R, et al.: Cognitive and problem solving training in children with cancer: a pilot project. *J Pediatr Hematol Oncol.* 2009;31(9):670–7. 19707159 10.1097/MPH.0b013e3181b25a1d

 With many thanks again for the thoughtful comments and review.
